# Quantitative Influenza Follow-Up Testing (QIFT)—A Novel Biomarker for the Monitoring of Disease Activity at the Point-of-Care

**DOI:** 10.1371/journal.pone.0092500

**Published:** 2014-03-21

**Authors:** Xi Chen, Kaveh Pouran Yousef, Susanne Duwe, Katharina Karsch, Sandeep Grover, Stephanie Wählisch, Patrick Obermeier, Franziska Tief, Susann Mühlhans, Lea Seeber, Max von Kleist, Brunhilde Schweiger, Barbara Rath

**Affiliations:** 1 Department of Paediatrics, Division of Pneumonology-Immunology, Charité University Medical Centre, Berlin, Germany; 2 AG systems Pharmacology & Disease Control, Department of Mathematics and Computer Science, Free University Berlin, Berlin, Germany; 3 Robert Koch Institute, Division 17 Influenza and Other Respiratory Viruses, National Reference Centre for Influenza, Berlin, Germany; CEA, France

## Abstract

**Background:**

Influenza infections induce considerable disease burden in young children. Biomarkers for the monitoring of disease activity at the point-of-care (POC) are currently lacking. Recent methodologies for fluorescence-based rapid testing have been developed to provide improved sensitivities with the initial diagnosis. The present study aims to explore the utility of second-generation rapid testing during longitudinal follow-up of influenza patients (Rapid Influenza Follow-up Testing = RIFT). Signal/control fluorescent readouts (Quantitative Influenza Follow-up Testing = QIFT) are evaluated as a potential biomarker for the monitoring of disease activity at the POC.

**Methods and Findings:**

RIFT (SOFIA) and QIFT were performed at the POC and compared to blinded RT-PCR at the National Reference Centre for Influenza. From 10/2011-4/2013, a total of 2048 paediatric cases were studied prospectively; 273 cases were PCR-confirmed for influenza. During follow-up, RIFT results turned negative either prior to PCR (68%), or simultaneously (30%). The first negative RIFT occurred after a median of 8 days with a median virus load (VL) of 5.6×10∧3 copies/ml and cycle threshold of 37, with no evidence of viral rebound. Binning analysis revealed that QIFT differentiated accurately between patients with low, medium and high viral titres. QIFT increase/decrease showed 88% agreement (sensitivity = 52%, specificity = 95%) with VL increase/decrease, respectively. QIFT-based viral clearance estimates showed similar values compared to PCR-based estimates. Variations in viral clearance rates were lower in treated compared to untreated patients. The study was limited by use of non-invasive, semi-quantitative nasopharyngeal samples. VL measurements below the limit of detection could not be quantified reliably.

**Conclusions:**

During follow-up, RIFT provides a first surrogate measure for influenza disease activity. A “switch” from positive to negative values may indicate a drop in viral load below a critical threshold, where rebound is no longer expected. QIFT may provide a useful tool for the monitoring of disease burden and viral clearance at the POC.

## Introduction

Influenza may cause significant morbidity and mortality, especially in infants and children [1]–[3], who tend to exhibit high viral loads and prolonged viral shedding [2], [4], [5]. In paediatric patients, influenza may also evoke a wide range of nonspecific symptoms, which are often difficult to predict or quantify [4],[6]. Much progress has been made with respect to rapid influenza diagnostic testing (RIDT), but standardized assessments for the longitudinal monitoring of influenza infections are currently lacking.

Ideally, influenza infections would be monitored in real-time, at the point-of-care (POC). Immediate assessments would be particularly important in the inpatient setting and during the peak of flu season, when infection control measures are increasingly challenging and many different physicians may be assessing the patient over time. In influenza patients receiving antiviral therapy, an objective POC measure of viral load should be able to discriminate treatment success from virologic failure, which may contribute to the emergence of drug resistance [7]. WHO and CDC guidelines recommend extending the duration of antiviral therapy beyond five days in influenza patients with evidence of ongoing viral replication, immuno-compromise or severe illness (i.e. ICU-level care) [8], [9]. Considering the possibility of antiviral resistance and viral rebound with premature termination of treatment, simple means of estimating viral clearance in high-risk patients are needed [7], [10]–[13]. Furthermore, clinical trials evaluating newly developed antivirals will require objective parameters to evaluate antiviral efficacy compared to, or in combination with, currently available drugs [12], [14].

Molecular methods and viral culture are the most commonly used methods for the assessment of viral shedding in influenza patients [15], [16]. These assays usually require considerable laboratory equipment and staff, and test results may take several days to become available to the physician [6], [15]. RIDT has been developed for the immediate diagnosis of influenza infections in the acute care setting [16]–[20]. RIDT are most sensitive with CT values <30 [21]. Second-generation RIDT (such as the SOFIA) use fluorescence-labelled antigens combined with POC readers, providing the advantage of standardized readouts. Evaluations in the US, Asia and Germany revealed improved sensitivities of up to 88% compared to RT-PCR with specificities close to 100% [21]–[24]. Rapid antigen tests reflect the actual amount of viral particles in a given sample, and tend to correlate well with viral culture results [22], [25]. PCR-based methods on the other hand, include an amplification step and are highly sensitive to the presence of nucleic acid. With the exception of capped m-RNA PCR, standard RT-PCR methods are not as specific for the presence of fully intact and infectious virus particles. An ideal POC test for the longitudinal follow-up of laboratory-confirmed influenza infections should therefore measure disease activity based on a critical amount of infective virus, even at the expense of lower sensitivities compared to PCR. Rapid antigen test may be able to fill this gap. During the process of viral clearance, the loss of antigen-positivity may indicate successful treatment or “overcoming” of natural infection, whereas RT-PCR results tend to remain positive for extended periods of time, even beyond resolution of clinical symptoms [7].

We hypothesized that *qualitative* (“positive”/“negative”) as well as *quantitative* (“signal over control”) luminescent readouts from second-generation rapid antigen tests may prove to be useful as an immediate estimate of virus burden and disease activity. We used SOFIA as an example of a simple rapid antigen test that can be performed by nurses or doctors at the bedside.

The present study aims to evaluate the utility of:

- “Rapid Influenza Follow-up Testing” (RIFT) for the (qualitative) monitoring of influenza infections over time as well as
- “Quantitative Influenza Follow-up Testing” (QIFT) as a potential biomarker for the monitoring of influenza disease activity (virus burden) at the POC.

## Methods

### Ethics Statement

Ethical approval was obtained from the Charité Institutional Review Board (EA 24/008/10). Informed consent procedures were waived for enhanced patient monitoring in the context of a quality management (QM) program.

### Study design and data collection

This prospective evaluation study was performed in the context of a QM program at the Charité Department of Paediatrics in collaboration with the National Reference Centre for Influenza at the Robert Koch Institute (RKI). From October 1, 2011 to April 30, 2013, all patients fulfilling pre-defined criteria for influenza-like illness (fever ≥38°C and ≥ one respiratory sign or symptom) were assessed consecutively by a specifically trained QM team [7], [21]. Cases were included if i) the patient was 0–18 years of age, and ii) influenza infection was confirmed by RT-PCR in the national reference laboratory. Cases were excluded from this analysis in case of: i) simultaneous infection with more than one influenza type or subtype [26], or ii) inability to obtain nasopharyngeal samples.

### Follow-up Testing

Members of a specifically trained QM team obtained nasopharyngeal samples, with rapid antigen tests (SOFIA) performed at the bedside. Patients with laboratory-confirmed influenza were scheduled for follow-up testing every two to three days, until at least one negative RIFT result was obtained or longer, if indicated. Basic clinical parameters and treatment with neuraminidase inhibitors were recorded. The respective clinician on duty made antiviral treatment decisions independently. With each rapid antigen test, blinded RT-PCR was performed in parallel at the RKI.

#### RIFT

Fluorescence-based RIFT was performed immediately at the POC and according to the manufacturer's protocol for the *SOFIA Influenza A+B FIA* test kit (Quidel Inc., San Diego, CA, USA). With each test, the SOFIA luminescent reader provided print-outs with qualitative test results (influenza A or B positive/negative), which were recorded and communicated without delay to the patient or parent as well as to the physician on duty.

#### QIFT

QIFT-values were calculated for each rapid antigen test based on the relationship between the influenza A and B test lines' fluorescence intensities and the cut-offs for each analyte with the SOFIA test. The resulting signal over control values (SCo) generated by the SOFIA POC reader ranged from 0 to 400. The limit of detection (LOD) with QIFT is 1; values >1 were considered positive.

#### RT-PCR and Viral Load (VL) Determination

Qualitative and quantitative RT-PCR were performed as described recently [7]. Briefly, nasopharyngeal swabs were washed out in cell culture medium and RNA was extracted either by using the MagAttract Viral RNA 48 Kit (Qiagen), the RTP RNA/DNA Virus Minikit (Invitek), or the MagnaPure 96 DNA and viral NA small volume kit (Roche). After random reverse transcription complementary DNA, primer and probes targeting the M, HA and NA genes were used for detection and further subtyping of influenza [27]. The RT-PCR cycle threshold (CT) values obtained for each influenza positive sample were analysed comparatively with serially diluted plasmid standards. All reactions were performed using the Light Cycler 480 real-time PCR system (Roche). CT values obtained with influenza negative samples were defined as 45 for mathematical analyses. The limit of detection for RT-PCR (LOD_PCR_) depends on the targeting gene and was determined to be 2–15 genome equivalents per reaction (95% detection probability) [27]. Regarding dilution factors due to sample preparation, RNA extraction, cDNA synthesis, and PCR performing, the limit of viral load quantitation (LOQ) was estimated to be 1000 copies/ml. This value was defined as LOD_VL_, below which the calibration curve is no longer valid and quantitative values are not reliable. Comparison of virus load assessments based on CT versus quantitative PCR at the National Reference Centre showed a correlation of 0.95 (Spearman, p-value<0.0001; Figure S1). With a high correlation between the two methods of VL assessment in the reference laboratory, it was decided to use CT for the QIFT/PCR correlation analyses, with the advantage of a lower LOD_PCR_ compared to LOD_VL_.

### Data Analysis

Data analysis and reporting were conducted in accordance with the STARD guidelines [28], wherever applicable. The STARD checklist is provided in the Supplementary Materials (Text S1). The STARD was chosen as the most appropriate standard available, even though the reported study pertains to longitudinal follow-up of patients with *established* diagnoses rather than diagnostic accuracy. The diagnostic accuracy including sensitivities and specificities of SOFIA RIDT in relation to PCR in the same setting has been published elsewhere [21].

#### RIFT compared to longitudinal PCR results

All patients with at least one positive RIFT and completed follow-up series were included in the analysis. Qualitative RIFT data for each patient during follow-up were compared to the corresponding CT value in the RT-PCR.

Three key time points were determined for each disease episode:

- T_MV_  =  Time point of maximum viral load/ lowest CT
- T_LP_  =  Time point of last positive RIFT
- T_FN_  =  Time point of first negative RIFT.

The respective time points T_MV_, T_LP_, and T_FN_ were plotted against the respective CT values at T_MV_, T_LP_, and T_FN._ The y-axis was inserted at the time point where the switch from T_LP_ to T_FN_ occurred ( = “Switch Day”).

#### RIFT compared to viral culture

Overall rates of agreement and kappa scores were calculated (Stata Version 11.2), comparing the time period required for RIFT and viral culture to become negative, respectively.

#### QIFT and clinical parameters

For correlation analysis between QIFT and clinical presentation, the following clinical parameters were chosen: a) maximum body temperature, b) CRP and c) presence of tachypnea (according to age-adjusted WHO criteria).

Correlation analyses for a) and b) were conducted using the Spearman rank correlation test. For analysis of c), a non-parametric test (Mann Whitney) was used.

#### QIFT /PCR Correlation Analysis

For QIFT/PCR correlation analysis, two correlation plots were generated:

- First, all values were included. Any QIFT values < LOD_QIFT_ were set equal to the LOD_QIFT_ as the most conservative estimate. Values  = 0 (i.e. virus not detectable by either method) were set to 0.
- A second analysis was performed excluding any values < LOD.

Correlation analyses between continuous variables were conducted using the Spearman rank correlation test.

#### Categorizing viral load based on QIFT

Binning analyses were performed for the categorization (“binning”) of median virus load in relation to different QIFT categories (negative, low, moderate, and high QIFT). Continuous variables were compared between different categories (“bins”) using non-parametric tests (Mann Whitney, Kruskal Wallis).

For comparison between QIFT and CT values, a “sliding window plot” was generated using MATLAB 7.14 (MathWorks Inc., Natick, MA): All simultaneous measurements of QIFT vs. CT were sorted by the respective QIFT in ascending order. Every 30 consecutive QIFT values denote a sliding window.

#### Estimating viral load kinetics based on QIFT and PCR

Lacking a reference standard for the assessment of viral shedding/antigen clearance over time, overall rates of agreement and kappa scores were calculated comparing QIFT increase or decrease to PCR increase or decrease over time [29]–[32]. Each sequence of 2 consecutive samples from the same patient was included in the analysis. A trend in viral load was defined as increase or decrease if rates of change in relation to the previous measurement in the same patient exceeded 10%.

#### Viral clearance dynamics with and without treatment

Viral clearance dynamics with and without treatment were computed for VL (CL_VL_) and QIFT (CL_QIFT_). All cases with at least two positive samples with both VL and QIFT measurements available ( = QIFT population) were included. In patients receiving antivirals, treatment had to be completed and baseline swabs had to be obtained within 24 hours of treatment initiation. Patient-specific CL_VL_ and CL_QIFT_ were estimated with calculated quantitative values even below LOD, using the optimization routine *lsqcurvefit* in MATLAB version 7.10 (MathWorks Inc., Natick, MA) and a weighted least-square criterion to minimize residual error. Details are provided in the Text S2.

## Results

### Study population

From October 1, 2011 to April 30, 2013, a total number of 2048 disease episodes were assessed and documented prospectively; of these, 278 (13.6%) cases were laboratory-confirmed for influenza by PCR. Three cases with dual influenza infection (A/A, A/B and B/B) and two patients unable to provide nasopharyngeal samples were excluded, leaving for analysis 273 cases of laboratory-confirmed influenza with serial swabs available. (Two patients experienced two independent disease episodes due to different influenza viruses, both were included as separate cases in the analysis.)

Case characteristics at the time of enrolment with each disease episode are displayed in Table 1. Among 273 influenza cases ( = total population) 55 were “late presenters”, i.e. patients who appeared in the emergency room late in the course of illness, after loss of their antigen-positive status in the RIFT, which usually requires intact viral particles (as opposed to nucleic acid) and higher viral loads compared to PCR. Overall, 178 patients completed the full follow-up program until at least one negative RIFT was obtained (RIFT population), resulting in an overall completion rate of 85%. For QIFT/PCR correlation and binning analyses, all 669 quantitative antigen measurements were included. A total of 210 cases fulfilled QIFT population criteria (availability of > = 2 positive samples with both, VL and QIFT measurements). Among these, 183 patients were without antiviral therapy, and 27 completed oseltamivir therapy. There was no significant correlation between the decision to use antivirals and either the number of symptoms, the duration of illness, or the presence of underlying conditions (p = 0.595, p = 0.781 and p = 0.797, respectively).

**Table 1:** Characteristics of eligible PCR- confirmed influenza cases.

Category	Subcategory	Total cases	Age <2 years	Age 2–5 years	Age >5 years
		(%)	(%)	(%)	(%)
**Total N**		273	82	96	95
**Gender**	male	152	47	49	56
		(55.7)	(58.3)	(51.0)	(58.9)
	female	121	35	47	39
		(44.3)	(42.7)	(49.0)	(41.1)
**Underlying condition**	pulmonary	26	7	7	12
		(9.5)	(8.5)	(7.3)	(12.6)
	cardiac	20	4	7	9
		(7.3)	(4.9)	(7.3)	(9.5)
	endocrine	13	1	4	8
		(4.8)	(1.2)	(4.2)	(8.4)
	hepatorenal	4	1	0	3
		(1.5)	(1.2)	(0)	(3.2)
	neurologic	19	3	6	10
		(7.0)	(3.7)	(6.3)	(10.5)
	immuno- suppression	9	0	2	7
		(3.3)	(0)	(2.1)	(7.4)
	hematologic	2	1	0	1
		(0.7)	(1.2)	(0)	(1.1)
	Prematurity^a^	33	13	13	7
		(12.1)	(15.9)	(13.5)	(7.4)
**Treatment with Oseltamivir**	treated	33	16	11	6
		(12.1)	(19.5)	(11.5)	(6.3)
	untreated	240	66	85	89
		(87.9)	(80.5)	(88.5)	(93.7)
**Influenza type/ subtype**	A(H1N1)pdm09	70	27	23	20
		(25.6)	(32.9)	(24.0)	(21.1)
	A(H3N2)	112	38	47	27
		(41.0)	(46.3)	(49.0)	(28.4)
	B	91	17	26	48
		(33.3)	(20.7)	(27.1)	(50.5)

a <37 gestation weeks.

### Data Analysis

#### RIFT results tend to turn negative prior to PCR

**Figure 1:**
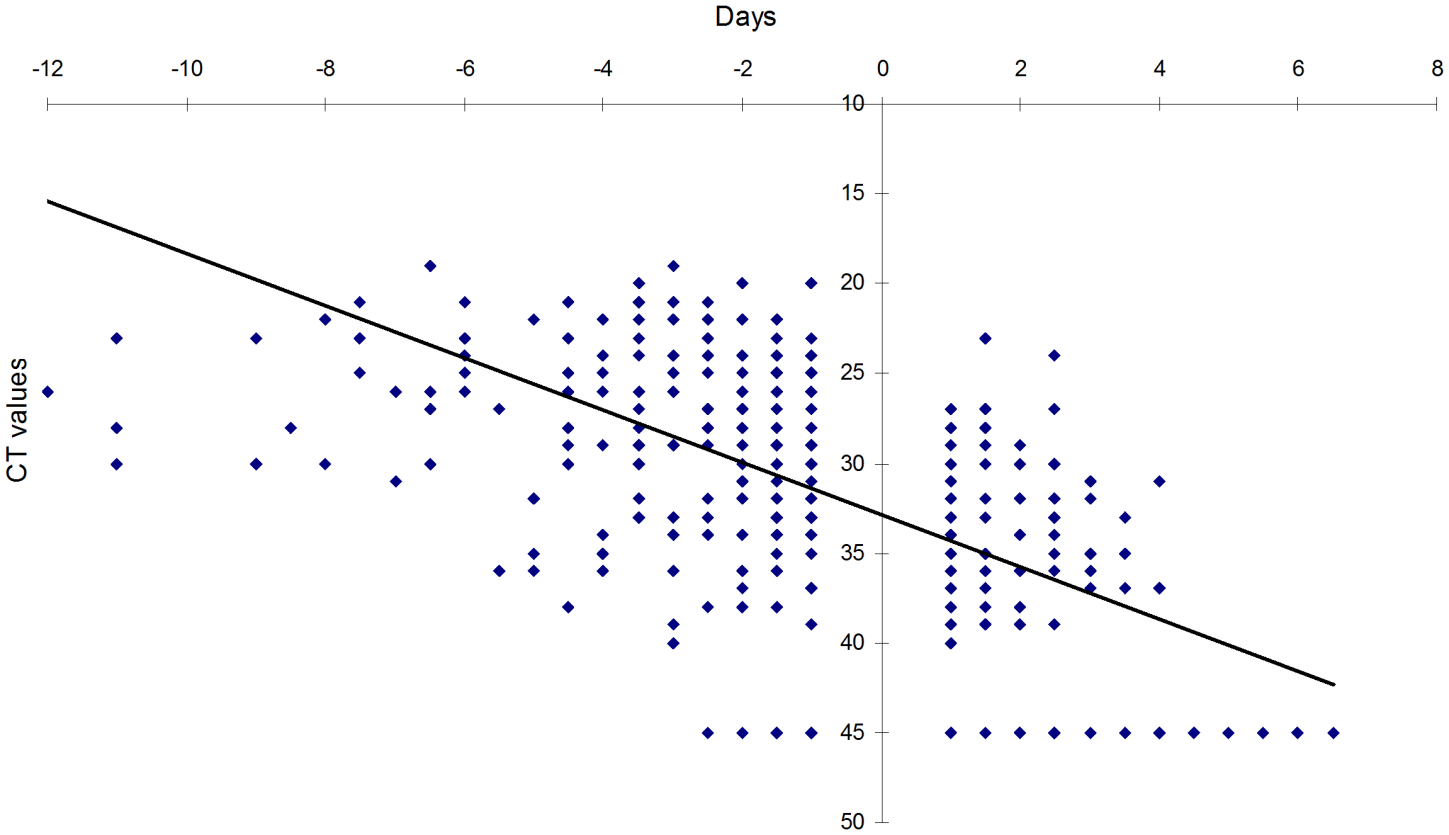
Comparison of CT values over time in relation to RIFT. The insertion of y-axis reflects the “switch” from positive to negative RIFT.

The qualitative results obtained with rapid influenza follow-up testing (RIFT) were compared to cycle thresholds with RT-PCR. The key follow-up time points T_MV_, T_LP_, and T_FN_ were determined for each disease episode and plotted against the corresponding CT values, as depicted in Figure 1.

During follow-up visits, the first negative RIFT was observed at a median of 8 days (range 2; 19) with a median CT of 37 (range 45; 23) and a median VL of 5.6×10∧3 (range 0; 78.4×10∧3). The first negative RIFT result was consistently observed prior to (68%) or simultaneous with (30%) a first negative RT-PCR result. There were only 4 instances (2%) where a positive RIFT was not confirmed by PCR, which was attributed to contamination of infant samples with RNAses. No cases of viral rebound were observed. The “Switch Day” from positive to negative RIFT, illustrated by insertion of the y-axis between T_LP_ and T_FN_, corresponded to a CT value of 33.

### RIFT results correlate well with virus culture

In 118 cases, at least two swabs with viral culture data in addition to RIFT were available, with a “switch” from positive RIFT and/or culture to negative RIFT and/or culture. Time required for RIFT to become negative from a previously positive value was 5.97±3.16 days; (range 2; 18).Time required for viral culture to become negative was 5.31±2.67 days (range 2; 14).

We observed an overall rate of agreement of 77.97% between the time period required for RIFT to become negative and time period required for viral culture to become negative (kappa = 0.75; p-value<0.0001). With respect to correlation analysis between time periods, we observed a Spearman's correlation of 0.803 between the two time periods (p-value<0.0001).

#### No significant correlation between QIFT values and clinical parameters

QIFT showed no significant correlation or association to the following clinical parameters: a) maximum body temperature (Spearman Rho = −0.027, p = 0.667), b) CRP (Spearman Rho = −0.087, p = 0.338) and c) presence of tachypnea (Mann Whitney, p = 0.154).

#### QIFT values close to the LOD correlate poorly with PCR

**Figure 2:**
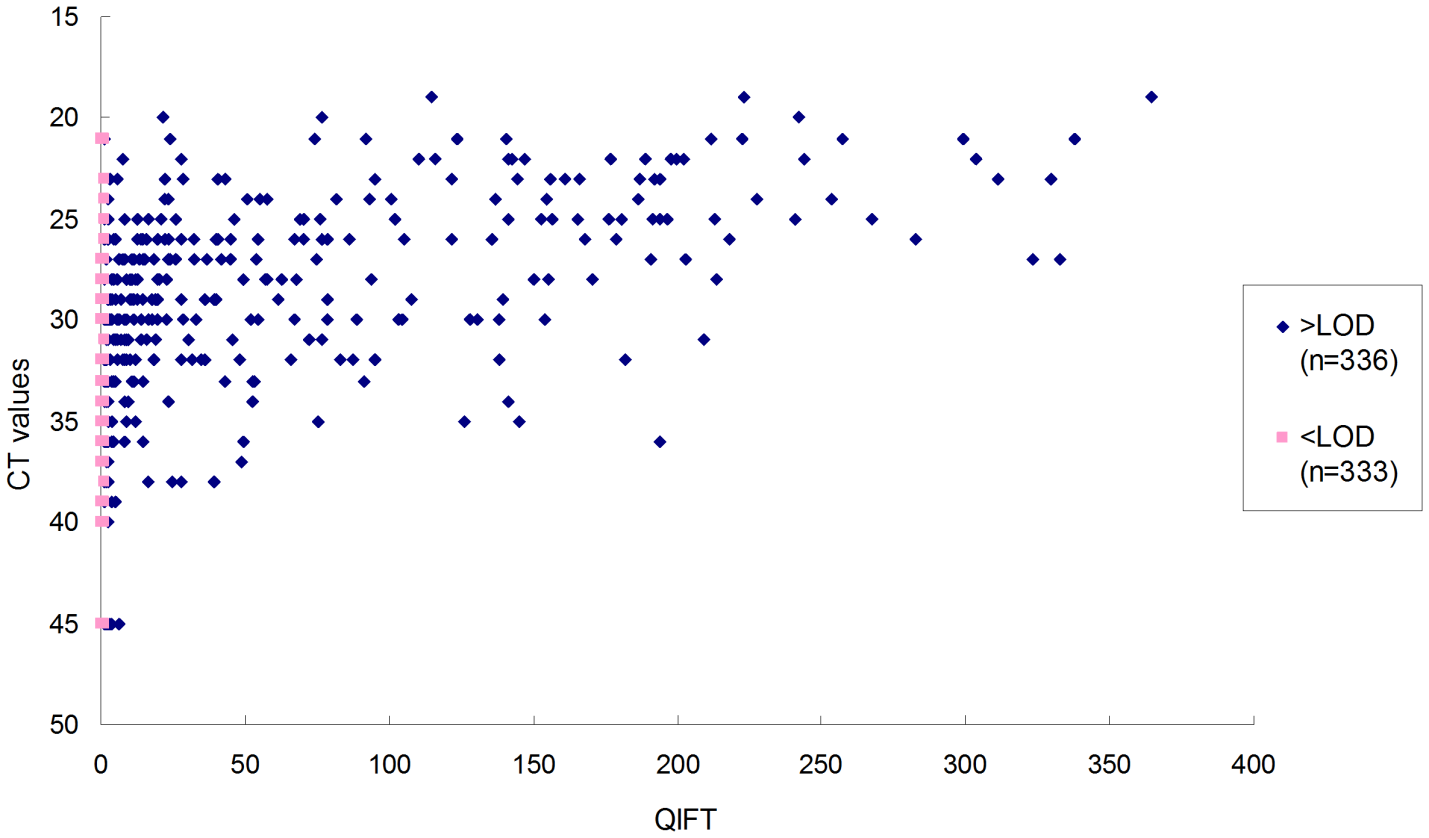
Comparison of CT versus QIFT. LOD_QIFT_ was defined as QIFT = 1.

All quantitative measurements obtained during longitudinal follow-up amounted to 669 measurements with QIFT and matching CT values. QIFT/PCR correlations are depicted in Figure 2. QIFT showed a significant correlation with CT of 0.69 (Spearman, p-value<0.0001) with QIFT values < LOD set to LOD  = 1 as the most conservative estimate. Following the RIFT observations illustrated above, it is expected that during longitudinal follow-up (of which all values are included in this plot), RIFT will turn negative prior to PCR. Figure 2 illustrates that QIFT values near or below the LOD_QIFT_ correlated poorly with PCR, compared to higher QIFT values.

#### Categorization based on QIFT provides useful estimate of virus burden

**Figure 3:**
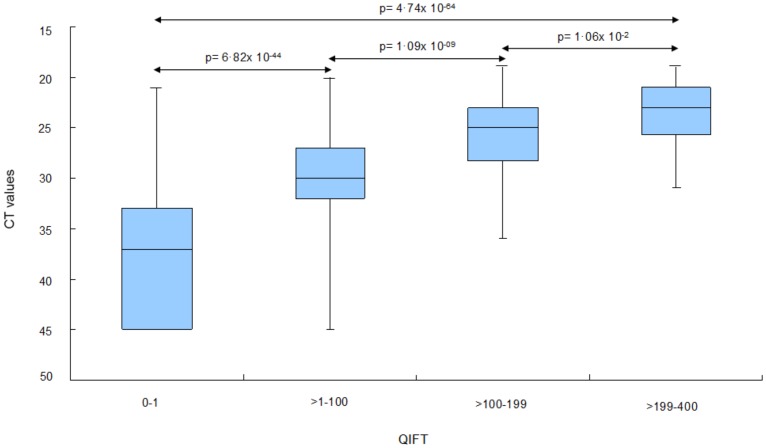
Binning analysis: Estimation of CT, median values based on categorized QIFT readouts. The following QIFT categories were used: “Negative QIFT”: 0 to 1 (n = 333). “Low QIFT”: >1 to 100 (n = 254). “Moderate QIFT”: >100 to 199 (n = 56). “High QIFT”: >199 (n = 26).

For the categorization of virus load based on low, moderate and high QIFT values, binning analyses were performed: QIFT readings were categorized into the following “bins”: “negative” (0–1), “low” (i.e. >1–100), “moderate” (>100–199), and “high” (>199–400). Median CT values differed significantly with respect to the four QIFT categories (Mann Whitney, Kruskal Wallis, p-value<0.0001), see Figure 3.

**Figure 4:**
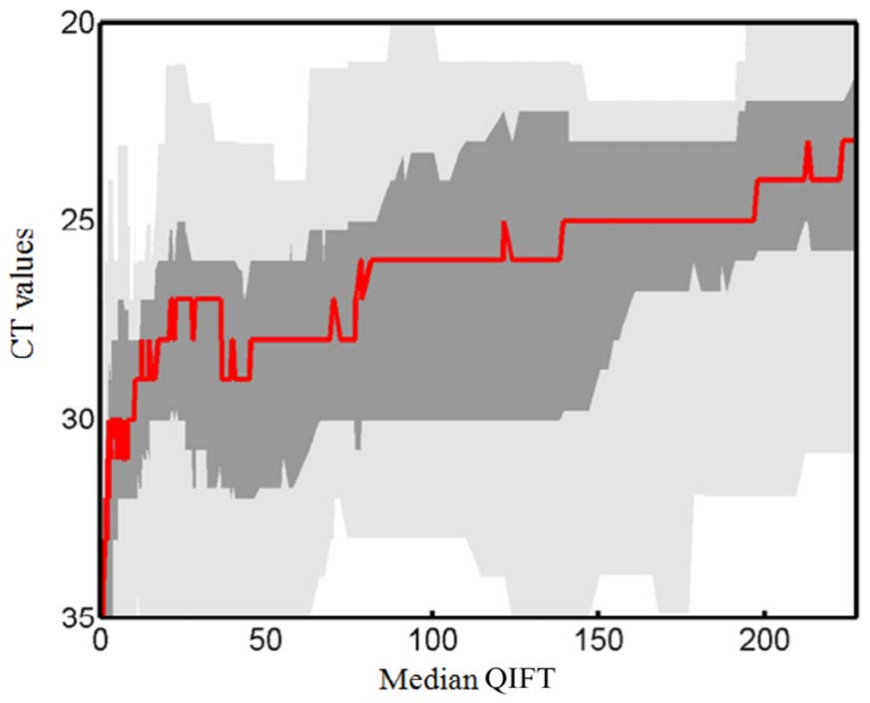
Sliding Window Graph: Binning of median CT values and ranges/percentiles based on median QIFT.

A Sliding Window Plot was generated to evaluate the performance of any particular QIFT in predicting CT values (Figure 4). The resulting graph illustrates a percentiles curve for the estimation of CT based on QIFT, including medians for the respective QIFT value. Please note that with 669 samples, there is a relatively broad coefficient of variation of approximately 20%.

#### Up- and downward trends in virus load are reflected similarly by PCR and QIFT

To determine trends in virus burden based on quantitative PCR versus QIFT, rates of agreement between “QIFT-increase”, “QIFT-decrease”, “VL-increase”, and “VL-decrease” were calculated. Overall agreement was 88% (kappa = 0.49; p-value<0.0001), see Table 2.

**Table 2:** Rates of agreement between QIFT and VL increase/decrease.

	QIFT ↑	QIFT ↓	
VL ↑	29	31	
VL ↓	16	320	
Rate of Agreement	**64%**	**91%**	**88%**

### Viral clearance rates in treated and untreated patients may be monitored using QIFT

**Figure 5:**
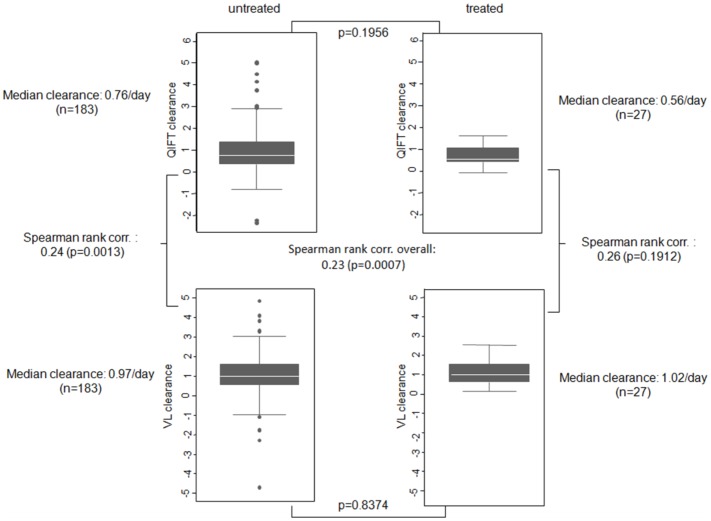
Comparison of viral clearance rates for CL_VL_ versus CL_QIFT_ and for treated versus untreated patients. Variance in viral clearance rates for quantitative is smaller in treated than in untreated patients, for both VL and QIFT.

Median clearance rates CL_VL_ and CL_QIFT_ in QIFT patients with and without antiviral treatment are depicted in Figure 5.

Overall, CL_QIFT_ showed slightly lower, but similar values compared to CL_VL_. In treated patients, median clearance rates were 1.02/day when determined by PCR (CL_VL_), and 0.56/day when measured by QIFT (CL_QIFT_) (Spearman: 0.26; p = 0.1912). In untreated cases, the median CL_VL_ was 0.97/day and median CL_QIFT_ was 0.76/day (Spearman: 0.24; p = 0.0013). In patients receiving antiviral therapy, viral clearance rates based on PCR (CL_VL_) showed less variability (range 0.08; 2.49) compared to untreated patients (range −4.70; 4.84). The same observation held true with respect to viral clearance rates based on antigen testing (CL_QIFT_) ranging from −0.07 to 1.62 and from −2.36 to 5.03 in treated *versus* untreated patients, respectively.

## Discussion

### Statement of Principal Findings

We report a first prospective study investigating “loss of antigen positivity” as a POC parameter for the longitudinal monitoring of influenza patients. During follow-up, rapid antigen testing using fluorescence-based laminar flow tests (RIFT) may provide a practical surrogate measure for influenza disease activity. “Loss of antigen positivity” in RIFT may indicate a drop in viral load below a critical threshold, where viral rebound is no longer expected. It may also indicate loss of infectivity as reflected by viral culture turning negative simultaneously with RIFT in most cases.

Quantitative readouts from the same rapid test (QIFT) show potential as a valuable biomarker for the monitoring of virus burden over time, with the advantage of obtaining results immediately at the POC. QIFT may also be able to discriminate between disease progression (VL increase) and resolution (VL decrease). In patients receiving antiviral therapy, QIFT can be used to monitor treatment success *versus* failure and/or risk of antiviral drug resistance by estimating viral clearance rates under therapy [7].

### Rapid Follow-up Testing in Context

For effective infection control measures and to reduce morbidity and mortality, isolation precautions are imminent. Antiviral treatment when indicated needs to be initiated as timely as possible [9], [33]. The need to diagnose treatable conditions with high disease burden has driven the development of diagnostic assays to be used at the POC. In contrast to first-generation RIDT, where sensitivities were still relatively low and user-dependent [34], second-generation RIDT combine improved sensitivities with objective automated read-outs [21], [22], [24].

Recent fluorescent-based RIDT such as SOFIA issue *qualitative* (positive/negative) results based on *qualitative* signal over control measurements. While RIDT have been licensed for the initial diagnosis of influenza at the POC [1], [6], [17], this pilot study demonstrates that *qualitative* (RIFT) and *quantitative* (QIFT) fluorescence-based antigen testing may both be useful for the longitudinal monitoring of influenza infections over time.

### Strengths and Limitations of the Study

The retention rate in this study was >85%, which is very high for an observational setting. For the evaluation of rapid influenza testing as a follow-up tool (RIFT) and QIFT-based clearance rates however, a sufficient number of follow-up time points was required. Hence, smaller subsets of patients were included in these analyses, representing 65% in RIFT and 80% in QIFT clearance rate calculations.

During longitudinal follow-up, RIFT turned negative consistently with, or after the PCR. It has to be borne in mind, that PCR and antigen tests measure different aspects of the same disease. While PCR-based (amplification) methodologies may be sensitive enough to detect even small amounts of nucleic acid over extended periods of time, direct antigen testing may provide an immediate (linear) estimate of virus burden during the acute phase of the disease only. It has been known that RIDT correlate better with viral culture than with PCR. It was therefore expected that the agreement between RIFT and PCR would decline during longitudinal follow-up (until both test are negative), whereas the duration of viral culture positivity may correlate well with antigen positivity [22], [25]. It is also to be expected that the lower sensitivity of rapid antigen testing compared to PCR would affect the QIFT/PCR correlation, especially later during the course of illness, when “loss of antigen positivity“ has already occurred and RIFT has turned negative ( = QIFT<1). VL measurements from PCR-positive samples below the LOD_QIFT_ could however not be quantified reliably. For utmost transparency, values below the LOD_QIFT_ were included in the analyses, but set to equal the highest possible value ( =  LOD) as the most conservative estimate. Reversely, negative QIFT corresponded well with negative PCR results.

QIFT and PCR both rely on the semi-quantitative measurement of virus load in nasopharyngeal samples – unlike in the case of HIV and hepatitis VL monitoring, where *plasma* virus loads are followed. To minimize variability with nasopharyngeal sampling, all RIFT/QIFT assays were performed immediately at the POC, and by a specifically trained QM team. Samples were delivered immediately to the RKI for blinded PCR analyses. The value of optimized sampling procedures has been demonstrated in previous evaluations establishing the sensitivity and specificity of the same assay (SOFIA for influenza A and B) in the same QM setting [21]. Use of QIFT and RIFT in less controlled settings may yield different results. Even if sampling has been optimized however, it must be noted that nasopharyngeal samples may not always reflect the virus burden present in the lower respiratory tract in critically ill patients, where VL may be elevated in patients infected with influenza viruses [11], [35]. Also, viral shedding in the lower airways is known to be prolonged in adult patients with pneumonia or immuno-compromise. In these patients, relapse of disease can occur if therapy is stopped based on cessation of upper airway replication alone [11]. This however, would require broncho-alveolar lavage procedures, which would neither be ethical nor feasible for the routine follow-up of paediatric influenza patients (unless intubated and critically ill). The present study does not allow for comparison of specific patient subpopulations. Further research in specific risk groups is needed.

Future analyses in specific subpopulations may compare the 3-way relationship between of PCR-positivity, antigen-positivity and viral culture in influenza patients with extreme disease presentations or different levels of immunosuppression.

### Future Perspectives

The results of the binning analyses demonstrate the practical value of QIFT for the monitoring of influenza disease activity at the POC. For QIFT to be used in clinical practice in the future, quantitative readings should be issued as four categories: negative, low, moderate, and high virus burden. QIFT as a follow-up tool could be optimized further, if the fluorescent reader provided a standardized display of trends in virus burden in relation to previous measurements in the same patient, or VL estimates as demonstrated in the binning and sliding window plots. Having immediate estimates of virus burden available at the POC will be of great value to support a personalized approach to the management of infants and immuno-compromised individuals with influenza infection who often exhibit elevated virus titres and prolonged virus shedding [2], [4], [5]. Previous studies have shown that virus burden cannot be estimated based on clinical appearance alone. It has further been reported that viral shedding may persist beyond cessation of clinical symptoms [4], [36]. Objective parameters such as RIFT and QIFT may facilitate individualized therapy of influenza with estimates of virus burden available immediately at the POC.

The decision whether or not to treat with antivirals in this QM setting, was left up to the clinician in charge. Interestingly, only a small proportion (12.1%) of patients with laboratory–confirmed influenza received neuraminidase inhibitor therapy. For all treated patients oseltamivir was prescribed. In the described setting, zanamivir is restricted to antiviral therapy in ICU patients or patients unable to take p.o. medicine. There was no evidence of correlation between treatment decision and patients' clinical symptoms, underlying conditions or late appearance to the hospital. With new antivirals under development, it might be of value to pursue QIFT as a follow-up tool in randomized controlled clinical trials.

Current recommendations by health authorities regarding established neuraminidase inhibitor therapies include a standard five-day course of treatment with a possible extension to ten days in cases of “ongoing viral replication” [8], [9]. Once antiviral therapy has been terminated, it should not be restarted. This implies that the decision whether to stop or adjust antiviral therapy has to be made on day five, at the POC. Relative under-dosing and/or premature treatment termination harbour the risk of promoting antiviral drug resistance [10], [13].

Previous studies have indicated that during antiviral therapy, a less-than-expected slope of decline in VL might indicate resistance development [7]. QIFT shows potential as a simple tool for the real-time monitoring of treatment success at the POC: QIFT-based viral clearance estimates (CL_QIFT_) showed similar ranges and percentiles compared to PCR-based VL estimates (CL_VL_). CL_VL_ however may be more accurate in lower ranges and with prolonged treatment. Further analyses in larger cohorts of treated influenza patients may be required to determine the exact relationship between CL_QIFT_ and CL_VL_. The interesting observation that antiviral treatment may reduce intra-individual variation in viral clearance rates with both CL_QIFT_ and CL_VL_ warrants further investigation.

Additional studies may be undertaken to investigate the cost-effectiveness of individualized antiviral therapy as well as the longitudinal monitoring of virus burden in infants, high risk and immuno-compromised individuals. The high retention rate in this QM program indicates that the availability follow-up data at the POC and the immediate feedback and reassurance to concerned patients and families may have a positive impact on patient satisfaction.

## Conclusions

RIFT and QIFT show promise as simple and objective tools for the real-time monitoring of influenza infections over time. RIFT may be used as an objective measure for “loss of antigen positivity”, whereas QIFT provides a useful estimate of viral clearance over time. The ability to monitor disease activity at the POC may have important implications for infection control. It may also facilitate the individualized management of infants and young children as well as immuno-compromised patients under antiviral therapy. Future studies in larger cohorts of patients will explore the practical implications of RIFT and QIFT in different clinical and demographic settings.

## Supporting Information

Figure S1 **Comparison of CT versus VL determined by RT-PCR.** LOD_VL_ was defined as VL = 1000 copies/ml. Spearman Rho = 0.95 (p-value<0.0001).
(TIF)

Text S1 **STARD checklist.**
(DOCX)

Text S2 **Calculation of viral clearances.**
(DOC)
