# Supplementary figures and images for: Quantitative Influenza Follow-Up Testing (QIFT)—A Novel Biomarker for the Monitoring of Disease Activity at the Point-of-Care
Source: PLoS One. 2014 Mar 21;9(3):e92500. doi: 10.1371/journal.pone.0092500 (PMC3962407; doi:10.1371/journal.pone.0092500)

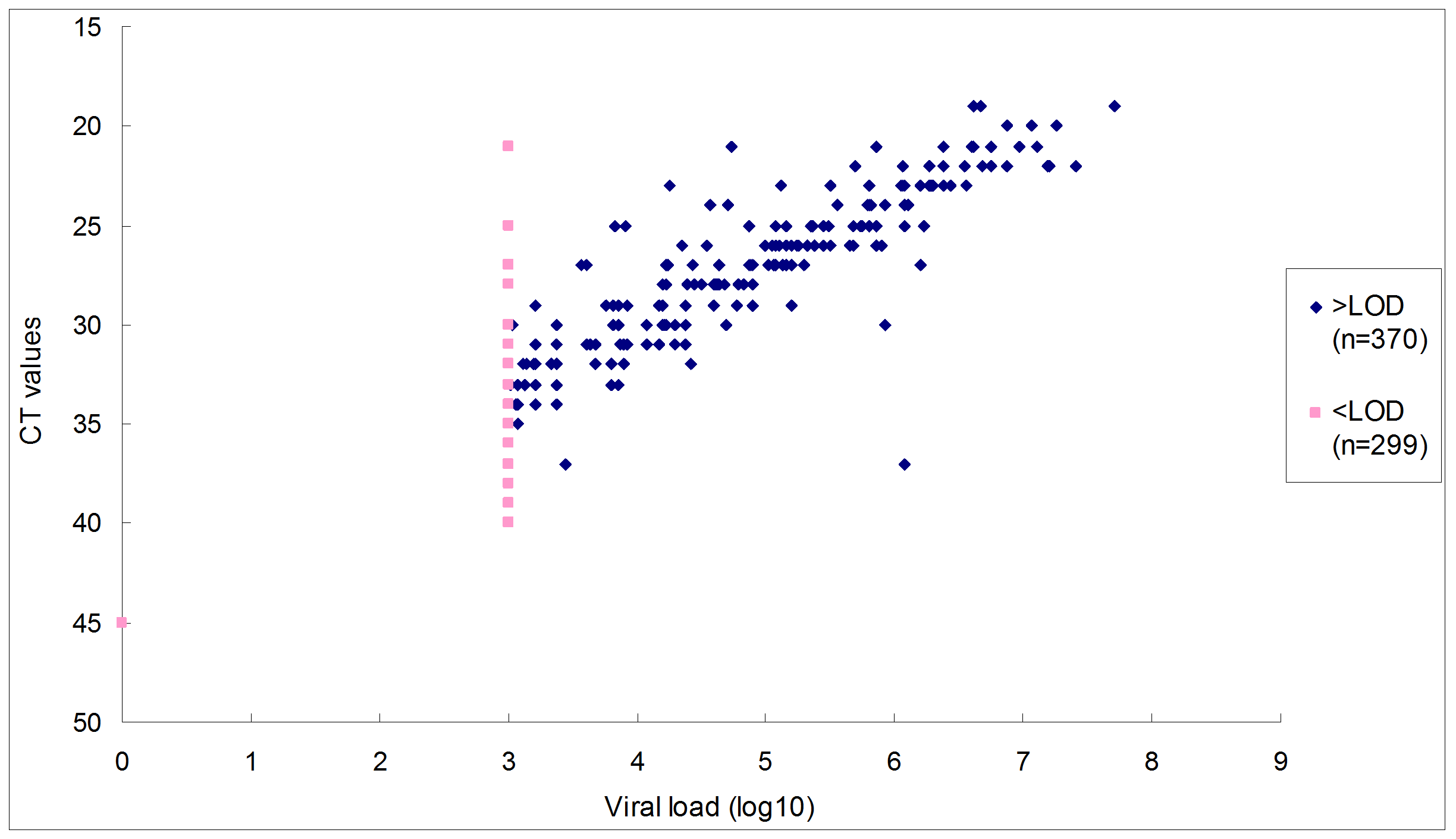

Supplement: Figure S1 — Comparison of CT versus VL determined by RT-PCR. LODVL was defined as VL = 1000 copies/ml. Spearman Rho = 0.95 (p-value<0.0001). (TIF) [file pone.0092500.s001.tif]
