# Supplementary material for: Quantitative Influenza Follow-Up Testing (QIFT)—A Novel Biomarker for the Monitoring of Disease Activity at the Point-of-Care
Source: PLoS One. 2014 Mar 21;9(3):e92500. doi: 10.1371/journal.pone.0092500 (PMC3962407; doi:10.1371/journal.pone.0092500)
Supplement: Text S2 — Calculation of viral clearances. (DOC) [file pone.0092500.s003.doc]

## Calculation of viral clearances

Patient specific clearance rates were estimated by the optimization routine *lsqcurvefit* in MATLAB version 7.10 (MathWorks Inc., Natick, MA), using a weighted least-squares criterion to minimize the residual error.

For the virus load based kinetics, we minimized the error between the data and model prediction according to:

where $\hat{V}L(i,t)$and $VL(i,t)$ denote the estimated and experimentally determined virus load in nasopharyngeal secretions of patient $i$ at time $t$after the first appearance in hospital. $\hat{V}L(i,t=0)$is the estimated initial virus load. This parameter was part of the estimation procedure to ensure that all patient values were equally weighted (the resulting estimates are not biased by initial values). $\hat{V}L(i,t)$was assumed to exhibit first-order clearance kinetics (with rate $CL_{VL}(i)$) according to:

For the QIDT based kinetics, we minimized the error between the data and model prediction according to:

where $Q\hat{I}FT(i,t)$and $QIFT(i,t)$denote the estimated and experimentally determined quantitative rapid test results of patient $i$ at time $t$ after the first appearance in hospital. $Q\hat{I}FT(i,t=0)$is the estimated initial QIDT. This parameter was also the part of the estimation procedure to ensure that all patient values were equally weighted (the resulting estimates are not biased by initial values). Likewise, $Q\hat{I}FT(i,t)$was assumed to exhibit first-order decay (with rate $CL_{QIFT}(i)$) according to:
